# A Comparison of the Quality of Informed Consent for Clinical Trials of an Experimental Hookworm Vaccine Conducted in Developed and Developing Countries

**DOI:** 10.1371/journal.pntd.0005327

**Published:** 2017-01-23

**Authors:** David J. Diemert, Lucas Lobato, Ashley Styczynski, Maria Zumer, Amanda Soares, Maria Flávia Gazzinelli

**Affiliations:** 1 Department of Microbiology, Immunology and Tropical Medicine, The George Washington University School of Medicine and Health Sciences Washington, DC, United States of America; 2 School of Nursing, Universidade Federal de Minas Gerais, Belo Horizonte, Minas Gerais, Brazil; 3 Department of Medicine, The George Washington University School of Medicine and Health Sciences Washington, DC, United States of America; Ministère de la Santé Publique et de la Lutte contre les Endémies, NIGER

## Abstract

Informed consent is one of the principal ethical requirements of conducting clinical research, regardless of the study setting. Breaches in the quality of the informed consent process are frequently described in reference to clinical trials conducted in developing countries, due to low levels of formal education, a lack of familiarity with biomedical research, and limited access to health services in these countries. However, few studies have directly compared the quality of the informed consent process in developed and developing countries using the same tool and in similar clinical trials. This study was conducted to compare the quality of the informed consent process of a series of clinical trials of an investigational hookworm vaccine that were performed in Brazil and the United States. A standardized questionnaire was used to assess the ethical quality of the informed consent process in a series of Phase 1 clinical trials of the *Na*-GST-1/Alhydrogel hookworm vaccine that were conducted in healthy adults in Brazil and the United States. In Brazil, the trial was conducted at two sites, one in the hookworm non-endemic urban area of Belo Horizonte, Minas, and one in the rural, resource-limited town of Americaninhas, both in the state of Minas Gerais; the American trial was conducted in Washington, DC. A 32-question survey was administered after the informed consent document was signed at each of the three trial sites; it assessed participants’ understanding of information about the study presented in the document as well as the voluntariness of their decision to participate. 105 participants completed the questionnaire: 63 in Americaninhas, 18 in Belo Horizonte, and 24 in Washington, DC. Overall knowledge about the trial was suboptimal: the mean number of correct answers to questions about study objectives, methods, duration, rights, and potential risks and benefits, was 45.6% in Americaninhas, 65.2% in Belo Horizonte, and 59.1% in Washington, DC. Although there was no difference in the rate of correct answers between participants in Belo Horizonte and Washington, DC, there was a significant gap between participants at these two locations compared to Americaninhas (p = 0.0002 and p = 0.0001, respectively), which had a lower percentage of correct answers. Attitudes towards participating in the clinical trial also differed by site: while approximately 40% had doubts about participating in Washington, DC and Belo Horizonte, only 1.5% had concerns in Americaninhas. Finally, in Belo Horizonte and Washington, high percentages cited a desire to help others as motivation for participating, whereas in Americaninhas, the most common reason for participating was personal interest (p = 0.001). Understanding of information about a Phase 1 clinical trial of an experimental hookworm vaccine following informed consent was suboptimal, regardless of study site. Although overall there were no differences in knowledge between Brazil and the US, a lower level of understanding about the trial was seen in participants at the rural, resource-limited Brazilian site. These findings demonstrate the need for educational interventions directed at potential clinical trial participants, both in developing and developed countries, in order to improve understanding of the informed consent document.

## Author Summary

Informed consent is an essential element of the ethical conduct of clinical trials of new vaccines, regardless of the study setting. However, the quality of informed consent is often suboptimal. Some research has suggested that the quality of the informed consent process may be reduced in resource-limited areas compared to developed country settings. To test this, we conducted a study of the quality of the informed consent process in two similar Phase 1 clinical trials of the *Na*-GST-1/Alhydrogel hookworm vaccine that were conducted in healthy adult volunteers in Brazil and in the United States. In Brazil, the trial was conducted at two sites, one a large urban area (Belo Horizonte), and the other a rural, resource-limited region of the state of Minas Gerais; in the United States, the trial was conducted in Washington, DC. A structured questionnaire was administered after the informed consent document was signed at each of the three clinical trial sites, which tested understanding about the information contained in the document and attitudes toward the volunteers’ participation in the clinical trial. The results indicate that there were no substantial differences between the overall quality of the informed consent obtained from participants in the United States and in Brazil. However, a significant association was found between the particular site where the trial was conducted and the quality of the informed consent process, with residents of the site in rural Brazil having the lowest percentage of correct answers on the informed consent questionnaire. The informed consent process should therefore take into account the specific characteristics of the population in which the trial is being conducted.

## Introduction

The principle of informed consent is internationally recognized as one of the essential elements of the ethical conduct of research involving human subjects [1, 2]. Within its ethical and legal foundations, obtaining informed consent has two specific objectives: to respect and promote the autonomy of research participants, and to protect the research subjects from possible harm or exploitation [3]. The informed consent process depends upon five criteria: the willingness to participate, the capacity to make a decision, disclosure of information, comprehension, and the decision to participate [4].

The quality of the informed consent process is determined primarily by the level of the study volunteers’ understanding and by the absence of coercion from the decision-making process [5, 6]. The findings of research indicate that, in general, there are gaps in individuals’ knowledge of various aspects of the clinical trials for which they are being consented, which may potentially impact their decision to participate [7–12]. In this sense, critics fear that for many clinical trials, the informed consent process may not be fully meeting its intended objectives [13].

Breaches in the informed consent process are frequently described in reference to clinical trials conducted in developing countries [14]. Low levels of formal education, a lack of familiarity with biomedical research, and limited access to health services in these countries have been associated with an inadequate informed consent [15–20]. A meta-analysis of the subject, however, demonstrates that this problem is not limited to developing countries, as several aspects of the informed consent process are poorly understood by participants in clinical trials both in developing *and* in developed countries [3]. In fact, one of the few studies dedicated to an empirical comparison of consent obtained in developed and developing countries revealed that there were no substantial differences in the participants’ knowledge between the two settings [21].

Furthermore, Mandava *et al* observed that the understanding of information about studies varies within both groups of volunteers and that to assume that clinical trials conducted in developing countries are less ethical than those conducted in developed countries is an oversimplification of an undoubtedly complex situation [11]. Most of the studies published to date on the informed consent process, however, have acknowledged limitations in their methodologies. Critically, the use of different measurement instruments has hindered comparison of results between different clinical trials. In this sense, these investigations have provided limited contributions to the discussion on the comparative quality of the consent process in developed and developing countries.

Given the methodological limitations of studies reported in the literature, we conducted an investigation to compare the quality of the informed consent process of a series of clinical trials performed in Brazil and the United States, using a standardized questionnaire. Our research sought to answer the following question: *can the ethical quality of the informed consent of participants in clinical trials carried out in developed countries be considered superior to that obtained in developing countries*? The authors hypothesize that there is no substantial difference in the quality of informed consent of research subjects living in developed and developing countries.

The justification for this study resides in the need to assess whether there is cause for concern regarding the protection of research participants in countries in which clinical trials are being conducted. The identification of differences and similarities between informed consent processes in developed and developing countries may aid in the implementation of specific strategies to protect participants in each research setting. Among these strategies, we can cite the need to support and inform institutional ethical review committees in their evaluations of the informed consent process of proposed clinical research.

## Methods

### Objective

A descriptive quantitative study with a cross-sectional design was conducted to compare the quality of the informed consent process of two Phase 1 clinical trials performed in Brazil and the United States, respectively, of the *Na*-GST-1/Alhydrogel hookworm vaccine that is being developed by the Sabin Vaccine Development Partnership [22]. The trials were carried out in the cities of Belo Horizonte and Americaninhas (Brazil) and in Washington, D.C. (United States of America).

In Brazil, a Phase 1 clinical trial was conducted between 2011 and 2014 of the safety and immunogenicity of *Na*-GST-1/Alhydrogel administered with or without the GLA-AF immunostimulant in healthy adults (protocol SVI-10-01, NCT01261130). The principal objective of this trial was to estimate the frequency of adverse events to the candidate hookworm vaccine. Vaccinations were conducted first in the hookworm non-endemic site of Belo Horizonte and then in the hookworm-endemic area of Americaninhas, to establish the vaccine’s safety in a hookworm-unexposed population before testing it in endemic areas.

A Phase 1 clinical trial of similar design was conducted of *Na*-GST-1/Alhydrogel administered with or without a different immunostimulant (a CPG oligodeoxynucleotide) in Washington, DC, starting in 2014 (protocol SVI-GST-03, NCT02143518). In both clinical trials, healthy adults (aged 18–45 years in Brazil and 18–50 years in the USA) were enrolled and vaccinated by intramuscular injection according to a 0, 2, and 4-month schedule.

### Study sites

In Brazil, the SVI-10-01 clinical trial was carried out in two separate centers: in Belo Horizonte and in Americaninhas, 556 km from Belo Horizonte. Americaninhas is a town of approximately 1500 residents located in the mostly rural municipality of Novo Oriente de Minas Gerais, in the Mucuri Valley, in the northeast part of the state of Minas Gerais. Belo Horizonte is the capital of Minas Gerais, with a population of 2,479,175 inhabitants and a human development index (HDI) of 0.81, which is considered very high [23]. On the other hand, Americaninhas is a region with low social indicators: it has an HDI of 0.60, the 6^th^-worst amongst Minas Gerais municipalities [24]. Low levels of formal education are a concern: 57.1% of its inhabitants are illiterate [24].

In the United States, the SVI-GST-03 clinical trial was conducted at the George Washington (GW) Medical Faculty Associates, a high-volume outpatient clinic affiliated with the GW hospital in the urban center of Washington, District of Columbia.

For both clinical trials, all participants underwent the informed consent interview and, if they decided to participate, signed an informed consent form (ICF) that had been approved by the ethical review committees of the Centro de Pesquisas René Rachou and the Brazilian federal Ministry of Health (for SVI-10-01), as well as the George Washington University (for both trials). The approved ICFs that were used for the trial in Brazil in Belo Horizonte (S1 ICF) and Americaninhas (S2 ICF), as well as the approved ICF that was used for the trial in Washington, DC (S3 ICF) are included as supporting information. The ICFs for the two trials differed primarily in the description of the different adjuvants that were used in the vaccine formulations (GLA-AF in Brazil *vs*. CPG in the United States) and in country-specific requirements such as the inclusion of language related to the Health Insurance Portability and Accountability Act in the United States. However, the ICFs were the same regarding the description of the study rationale and the nature of the hookworm vaccine, the risks and benefits of the *Na*-GST-1/Alhydrogel hookworm vaccine, the number of vaccinations to be administered, the duration of the study (per participant), the type of procedures to be conducted, the fact that participation was voluntary, and that consent could be withdrawn at any time with no negative consequences to participants. Completion of the informed consent questionnaire was optional and was not required for participation in the rest of the respective study.

### Data collection

Data were collected through the use of a semi-structured questionnaire consisting of 32 questions that assessed the participants’ understanding of the information about the study presented in the informed consent document for the respective clinical trial as well as the voluntariness of their decision to participate. The questions sought to evaluate their knowledge of the purpose of the clinical trial, the study methods, the duration of the trial, the participants’ rights, and the potential risks and benefits of participation. The participants’ socio-demographic and economic information were also collected.

Given the lack of appropriate existing questionnaires for this type of clinical trial, especially in two languages, questions, although not pre-tested, were based on published questionnaires used for clinical studies for other disease areas [25–27]; the International Ethical Guidelines for Biomedical Research [1]; and, on the experience the researchers have gained from working in this field for over 13 years.

In order to improve understanding of the questions, the authors followed the recommendations of Vieira, which included using plain and easily understandable language (assessed by the Flesch reading-ease score); using general language rather than technical terminology; and, avoiding negative phrases and words with double meaning [28]. The questionnaire was originally formulated in Brazilian Portuguese and later translated into English by experts on the research subject. With the agreement of the researchers involved, this process favored an interpretation of concepts rather than a literal translation of terms. Questions were also tailored to the specific goals, risks and benefits of each clinical trial.

Given the need for reliability of the measuring instrument, the researchers opted for using open-ended questions since pre-determined answers to close-ended questions might influence the participants’ responses [28]. The preference for this type of question arises from a study by Lindegger *et al*, which revealed that the participants’ understanding of the informed consent information was overestimated when evaluated by instruments using close-ended questions compared with those using open-ended questions [26].

Data were collected after all volunteers participated in the informed consent process and signed the informed consent form. In Brazil, the questionnaire was administered by interviewers who had received training in how to standardize data collection and improve reliability, in order to minimize the risk of information bias. The interviewers were undergraduate and graduate students in nursing, education, psychology and medicine, had no relationship with the clinical research staff, and were specially trained to comprehensively transcribe the participants' responses.

In the USA, the questionnaires were self-administered at the study site clinic. Different methods of applying the questionnaire in the USA and Brazil were chosen due to differences in the level of education of the volunteers, as had been observed in previous studies carried out in the same areas. The administration of the questionnaire lasted on average 10 minutes and it was carried out at the same setting in which the informed consent process was conducted. Most of the questionnaires were completed on the day of first vaccination or, in a small number of cases at all sites, immediately after signing the informed consent form.

### Data analysis

After collection, data were coded and entered into an SPSS database (version 14.0) and Microsoft Excel. In order to ensure reliability, data were independently entered twice. In cases of discrepancy between the two entries, the lead researchers referred to the original questionnaire and determined the actual response by consensus.

Analysis of the open questions followed the categorization of the responses, based on the criterion of appearance frequency. To avoid bias in the process of categorization, this step was performed independently by two different professionals. The end results of this stage were compared; in cases of disagreement between the categorizations, the professionals debated, each justifying their choice. After an agreement had been reached by consensus, the participant’s response was classified into the appropriate category.

Data were initially analyzed using descriptive statistics including frequency calculations (simple and relative), as well as mean and standard deviation. Subsequent analyses compared the percentages of correct answers (categorical variables) using the chi-square test. A Knowledge Index (KI) was created to measure participants' knowledge on all issues evaluated. This index consists of the sum of the participants’ correct responses divided by the total number of questions (11) and is expressed as a percentage ranging from 0% (the participant answered all questions incorrectly) to 100% (the participant answered all questions correctly). The analysis variable was analysed by calculating the mean, median and interquartile ranges. The KI was compared between study sites using the one-way ANOVA and Tukey-HSD tests. A significance level (p value) of 0.05 was used for all analyses. The normality of continuous variables was assessed by the Kolmogorov-Smirnov test.

## Results

A total of 105 study participants completed the informed consent questionnaire and were included in the analysis: 63 (60%) from Americaninhas, 18 (17%) from Belo Horizonte and 24 (23%) from Washington, DC. In Americaninhas, 3 of 66 (4.5%) participants enrolled in the clinical trial declined to complete the questionnaire, whereas 12 of 36 (33%) and 0 of 24 (0%) declined in Belo Horizonte and Washington, DC, respectively. Significantly more participants in Belo Horizonte declined to complete the questionnaire than in either Americaninhas or Washington, DC (p = 0.016), although the reasons for refusal were not recorded.

The average age of those completing the questionnaire was 29.3 years (SD 8.9, range 18 to 50), which varied significantly by study site (Belo Horizonte, 23.7 years, Americaninhas, 29.6 years, Washington DC, 32.8 years; p = 0.021, Kruskal-Wallis test). The proportion of study participants who were female (46.7%), on the other hand, did not vary significantly between the study sites. Regarding the maximum level of education achieved by participants, 37 (35.2%) had primary education, 33 (31.4%) had secondary education, 25 (23.8%) had post-secondary education and 6 (5.7%) had post-graduate education; 4 (3.8%) participants were deemed illiterate (all in Americaninhas). Levels of education were not uniform across the study sites: the chi-square test revealed a statistically significant difference between the site of the clinical trial and the participants’ maximum level of education, with the lowest levels of education observed in Americaninhas (p<0.001). The distribution of the participants according to their level of education is shown in Table 1.

**Table 1:** Maximum level of education attained by study participants who completed the questionnaire, by study site.

Maximum Level of Education	Study Site						Total
	Americaninhas		Belo Horizonte		Washington		
	N	%	N	%	N	%	
Illiterate	4	6.4	0	0	0	0	4
Primary education	36	57.1	1	5.6	0	0	37
Secondary education	22	34.9	5	27.8	6	25.0	33
Post-secondary education	1	1.6	11	61.1	13	54.2	25
Post-graduate education	0	0	1	5.6	5	20.8	6
**Total**	**63**	**100.0**	**18**	**100.0**	**24**	**100.0**	**105**

Most participants did not have a health insurance plan (n = 77; 73.3%), had never participated in a clinical trial (n = 78; 74.3%), and had no formal employment contract (n = 72, 68.6%). The chi-square and Kruskal-Wallis tests revealed statistically significant differences between the location of the clinical trial and having a health insurance plan (p<0.001), or having previously participated in a clinical trial (p = 0.030). Regarding health insurance, it appears that only three participants had formal insurance in Americaninhas (4.0%), with higher proportions being found in Belo Horizonte (16.6%) and Washington, DC (79.1%). In terms of formal employment, most participants in Americaninhas (71.3%) and in Washington, DC (79.1%) had jobs, with a lower proportion in Belo Horizonte (44.4%). While 55.5% of participants in Washington, DC had previously participated in a clinical trial, much lower rates were seen among the participants in either Belo Horizonte (0.5%) or in Americaninhas (25.5%).

Table 2 shows the absolute and relative frequencies of correct answers to questions that evaluated participants’ knowledge regarding information about the clinical trial that was contained in the informed consent form. A majority of participants knew the correct answers to at least seven out of the eleven questions. The research subjects from Belo Horizonte had the highest percentage of correct answers, with an average of five questions (45%) answered correctly, followed by four in the United States (36%), and one in Americaninhas (19%).

**Table 2:** Distribution of absolute and relative frequencies of correct answers related to the knowledge of participants about information contained in the informed consent form.

	Study Site							
	Amer		BH		Wash			
Question	N	%	N	%	N	%	*X*²	P
What is the main goal of this study?	25	39.7	13	72.2	20	87.0	15.9	0.001
Why is the vaccine being tested?	31	49.2	12	66.7	14	58.3	1.95	0.382
How long will the study last?	0	0	1	5.6	0	0.0	4.88	0.087
If someone doesn’t participate what can happen to them?	32	51.6	15	83.3	20	87.0	12.42	0.002
What are the risks of participating?	17	27.0	12	66.7	9	36.3	6.57	0.037
If you get sick during the study what are you supposed to do?	43	68.3	12	66.7	20	87.0	4.44	0.349
Could there be adverse reactions that the doctors don't know about?	34	54	18	100	21	87.5	18.47	0.001
What are the side effects?	21	40.4	9	50.0	7	31.8	1.36	0.506
Can you contact someone if you have questions or doubts about the study?*	61	96.8	17	100	23	100	1.29	0.523
Will you be able to get medical care from the study team after the end of the study?*	40	64.5	7	38.9	9	40.9	5.91	0.052
What are the benefits of your participation in the research?^a^*	28	65.5	13	92.9	15	78.9^a^	4.55	0.153
**Total**	**63**	**100**	**18**	**100**	**24**	**100**		

Wash, Washington, DC; BH, Belo Horizonte; Amer, Americaninhas.

^a^ In the USA, the response “monetary incentive” was deemed acceptable, but not in Brazil given that provision of monetary compensation to clinical trial participants is forbidden by law in that country.

* Question had a lower absolute frequency of responses.

The analysis of each question demonstrated that the majority of participants understood that deciding not to participate in the clinical trial for which they were being asked to volunteer would not result in any negative consequences. However, in Americaninhas only 51% recognized that declining to participate was not associated with of any negative consequences of not participating, compared to 83% and 87% in USA and Belo Horizonte, respectively. In addition, high proportions were aware of what they should do in case of illness during the trial, the possibility that they might experience anticipated or unanticipated adverse effects after being vaccinated, and that they could contact members of the study team if they had any doubts or questions about the trial. As shown in Table 2, there were statistically significant differences between the study sites in the comprehension of the following items: the study objectives (p = 0.001); the consequences of choosing not to participate in the study (p = 0.002); the potential risks of the investigational vaccine (p = 0.037); and the possibility of unanticipated adverse effects (p = 0.001).

Table 3 summarizes the participants’ knowledge about information contained in the informed consent form by calculating a “knowledge index” (KI) consisting of the mean number of correct answers to these questions on the questionnaire, by study site. In Americaninhas, participants had an average of 45.9% of correct answers; in Belo Horizonte, 65.2%; while in Washington, DC, the percentage was 59.1%.

**Table 3:** Knowledge index according to study site.

Statistic	Americaninhas (%)	Belo Horizonte (%)	Washington, DC (%)
Average	45.9	65.2	59.1
Median	45.5	63.6	63.6
Mode	45.5	72.7	63.6
Standard deviation	17.4	10.9	14.2
Minimum	18.2	45.5	27.3
Maximum	90.9	81.8	81.8

Table 4 provides comparisons between the average number of correct answers in the KI according to the one-way ANOVA and Tukey-HSD tests. The overall association between the study site and the average knowledge about the clinical trial was statistically significant (one-way ANOVA: F = 13.931, p = 0.0001). Although there was no difference in the rate of correct answers between participants in Belo Horizonte and those in Washington, DC (p = 0.437), there was a significant gap between the KI of participants at these two locations compared to Americaninhas (p = 0.0002 and p = 0.0001, respectively), where a lower percentage of correct answers was recorded.

**Table 4:** Comparison of the mean Knowledge Index (KI) between study sites.

Study Site	Comparison between Study Sites	Mean Difference in KI	P*	95% CI	
				Minimum	Maximum
Americaninhas	BH	19.3	0.000	9.2	29.3
	Washington, DC	13.2	0.002	4.2	22.2
BH	Americaninhas	19.3	0.000	9.2	29.3
	Washington, DC	6.1	0.437	5.6	17.8
Washington, DC	Americaninhas	13.2	0.002	4.2	22.2
	BH	6.1	0.437	5.6	17.8

KI, Knowledge Index; CI, Confidence interval; BH, Belo Horizonte.

* Tukey-HSD Test.

Table 5 provides the absolute and relative frequencies of responses concerning the participants’ attitudes towards clinical research and the voluntariness of their decision to participate in the clinical trial. In all three locations, the majority of participants reported not being afraid of participating in the research and trusting the investigators responsible for the trial. However, in Belo Horizonte, 72.2% of participants declared having enrolled in the study only for the benefits, a situation that was not observed at the two other sites.

**Table 5:** Absolute and relative frequencies of responses concerning participants’ attitudes towards the clinical trial.

	Study Site							
	Amer		BH		Wash			
Question	N	%*	N	%*	N	%*	X²	P
When you signed the Informed Consent Form were you sure that you understood it?	59	96.7	18	100	24	100	2.77	0.25
Did you enter the study only for the benefits?	23	36.5	13	72.2	5	20.8	7.90	0.02
If these benefits didn't exist would you still enter the study?	22	34.9	8	44.4	14	58.3	2.39	0.30
Did someone influence your decision to enter the study?	12	19.5	2	11.1	3	12.5	10.47	0.01
Do you have any doubts about participating in the study?	1	1.5	7	38.9	10	41.7	26.88	0.00
Even without receiving any information would you enter the study?	10	15.9	1	5.6	2	8.3	1.72	0.42
Do you trust the study team/doctor?	63	100	17	94.4	24	100	4.88	0.09
If a study presents a risk to your life, would you still take part?	8	12.7	7	29.2	9	37.8	9.23	0.01
Do you believe that by participating in the study your health can improve?	57	90.5	5	27.7	6	25.0	47.75	0.00
Can the doctor decide for you whether to enter the study?	19	30.2	8	44.4	7	29.2	1.45	0.48
Were you afraid to participate in the study at any time?	24	38.1	5	27.7	1	4.2	9.36	0.01
Can another person decide for you to enter the study?	5	0.1	0	0	3	12.5	2.20	0.33
Did the meetings with the study team influence your decision to enter the study?	48	76.2	14	77.8	10	41.7	10.46	0.00

* Percentage who responded “yes” to the question.

Regarding the participants’ doubts about participating in the clinical trial, whereas in Washington, DC, and Belo Horizonte approximately 40% reported having doubts, in Americaninhas only 1.5% admitted having some concerns about participating. Virtually all participants (90.5%) resident at that site believed that participation in the clinical trial could lead to improvements in their health. In Washington, DC, only 4.2% of participants admitted to being afraid to participate, while the comparable values in Americaninhas and in Belo Horizonte were higher, at 38.1% and 27.7%, respectively (p = 0.01). It appears that at the Brazilian study sites, participation in informational meetings about the study with study team members was a significant factor that influenced their decision to participate (76.2% and 77.8% in Americaninhas and Belo Horizonte, respectively), while in Washington only 41.7% cited this influence (p = 0.001) (Table 5).

Table 6 details participants’ responses regarding their attitudes to the clinical trial using the Likert scale. In both Brazil and the United States, most (90.4% and 100%, respectively) respondents agreed or strongly agreed when asked if they agreed or disagreed with the statement, “You want to participate in the study.” When asked if they only “tolerated” participation in the clinical trial, most subjects in the United States and in Belo Horizonte disagreed or strongly disagreed. On the other hand, in Americaninhas, most participants agreed or strongly agreed with the same question (p = 0.02).

**Table 6:** Participants’ responses regarding attitudes about participating in the clinical trial, according to the likert scale.

		Study Site							
		Americaninhas		Belo Horizonte		Washington			
Statement	Response	N	%	N	%	N	%	*X*^2^	P
You only tolerate participation in the study	SD	16	25.8	7	38.8	10	40.0	18.99	0.02
	D	9	14.5	9	50.0	10	40.0		
	NAD	6	9.2	-		3	12.0		
	A	16	25.8	2	11.1	2	8.0		
	SA	15	24.2	-	-	-	-		
You want to participate in the study	SD	-	-	-	-	0	0	11.14	0.08
	D	3	4.7	-	-	-	-		
	NAD	3	4.7	1	5.5	-	-		
	A	28	44.4	7	38.3	4	16.6		
	SA	29	46.0	10	55.5	20	83.4		

SD, Strongly Disagree; D, Disagree; NAD, neither Agree nor Disagree, A: Agree, SA: Strongly Agree.

Table 7 shows the absolute and relative frequencies of the study subjects’ motivations for participating in the clinical trial. When asked about their main motivations for participating, those in Belo Horizonte and Americaninhas reported personal or social advantages that would benefit them. In contrast, of the study participants in Washington, DC, 17.4% said that participating in the trial would bring more benefits mainly to society through development of a new vaccine for hookworm. In Americaninhas, the most common reason for participating was motivated by personal interest; in Belo Horizonte, half of participants reported that their main reason for participating was a desire to help others. In the United States, high percentages cited the possibility of helping others and receiving monetary compensation as reasons for participating. The reasons for participating in the clinical trial varied significantly by study site (p = 0.001, chi-square test).

**Table 7:** Distribution of absolute and relative frequencies regarding the study subjects’ reasons for participating in the clinical trial.

	Study Site							
	Americaninhas		Belo Horizonte		Washington, DC			
	N	%	N	%	N	%	*X*^2^	P
**Reason for Participating**							88.9	0.001*
Personal decision [without the influence of others]	16	25.4	7	38.9	0	-		
To improving my health	15	23.8	0	-	0	-		
To benefit the world/community	11	17.5	9	50.0	6	26.1		
To receive medical care	11	17.5	0	-	0	-		
To help produce a vaccine	9	14.3	2	11.1	1	4.4		
To help others and produce a vaccine	0	-	0	-	4	17.4		
To help others and to receive monetary compensation	0	-	0	-	8	34.8		
Don’t know	1	1.6	0	-	4	17.4		

* Chi-square and Fisher's exact test.

## Discussion

The results of the study reported herein indicate that there were no substantial differences between the overall quality of the informed consent obtained from participants in similar clinical trials conducted in the United States, a developed country, and in Brazil, a developing one. Such a conclusion is supported by the absence of any statistically significant differences between participants in Belo Horizonte and the United States in their knowledge of information about the clinical trial contained in the informed consent form. However, our research nevertheless showed a significant association between the particular site where the trial was conducted and the quality of the informed consent process: statistically significant differences were observed in the study participants’ knowledge about the trial between Americaninhas, Belo Horizonte and Washington, DC, with residents of Americaninhas having the lowest percentage of correct answers on the informed consent questionnaire.

The inequality of living conditions within the Brazilian population is a widespread reality. In many regions of Brazil, substantial disparities exist between urban and rural areas with regard to household income, basic infrastructure, access to healthcare, and quality of education. For example, the municipality of Padre Paraíso, the seat of the Americaninhas district where one of the clinical trials of this study took place, has an HDI of 0.60 that is classified as low, whereas in Belo Horizonte, the capital of the state of Minas Gerais, the HDI is considered “very high” at 0.81 [24]. Socio-demographic and economic characteristics such as advanced age, low level of education, female gender, and low socioeconomic status have been associated with a reduced quality of the informed consent process [29–33]. Therefore, it is essential that the characteristics of the potential research participants being recruited into a clinical trial be adequately analyzed in order to identify factors that may negatively influence the quality of the informed consent obtained from them.

These characteristics must also be analyzed to support researchers in developing strategies to encourage the dissemination and understanding of information about clinical research, such as the use of appropriate language in the informed consent form and the development of more relevant educational interventions that match the context of study participants. There are suggestions in the literature that efforts to establish greater links between researchers and participants, such as creating an atmosphere of openness to dialogue and giving opportunities for asking questions, can facilitate the consent process [34].

Regarding the lower percentage of success on the informed consent questionnaire observed in Americaninhas, the relative lack of understanding at this site about the scientific purpose of the study suggests that the study subjects in Americaninhas did not consider themselves to be participants in clinical research. Instead, they may have conflated the scientific purpose of the clinical trial with the provision of medical care, a phenomenon that has been termed the “therapeutic misconception” [33]. Although the study volunteers were participating in a Phase 1 trial that by definition may not provide any direct benefit to participants, this phenomenon may have resulted from the fact that they either received the hepatitis B vaccine as the comparator vaccine or were offered it at the end of the study and, if necessary, received treatment for hookworm and anemia, as well as being referred for further investigations or management in cases of other illnesses or medical conditions. Commonly observed amongst clinical trial participants who are socially and economically disadvantaged, the therapeutic misconception results from confusion between routine medical care provided to study participants in the context of a trial and the objectives of the study in which an experimental product is being tested. Instead of understanding that they are participating in an experiment, these individuals believe that the research protocols are tailor-made for them and their health-related issues [35, 36].

This phenomenon was observed in a previous study conducted in Americaninhas, in which many participants in a clinical trial of the *Na*-ASP-2 vaccine against hookworm believed that the purpose of the investigation was the medical treatment of its participants rather than the testing of an experimental preventative vaccine [7]. The manifestation of the therapeutic misconception is in conflict with the doctrine of informed consent since the participant experiencing such a misconception may not adequately weigh the risks and benefits of their participation in the clinical trial and instead base their decision to participate on incorrect criteria and false expectations [37].

The limited knowledge of the consequences of not participating in a clinical trial that was observed in Americaninhas is similar to the findings of Tam *et al* [3], which demonstrated that participants in clinical trials conducted in developing countries are less acquainted with issues related to participation or refusal of participation in a study. Many studies have affirmed that information about the right to refuse participation and to withdraw consent at any time, without affecting their rights or access to medical care is one of the most important items to be conveyed to prospective research participants, particularly to those in developing countries [38, 39].

Despite the above differences, it should be noted that most participants—at all study sites—had an incomplete knowledge of the information contained in the informed consent form. The highest average knowledge as assessed by a structured questionnaire amongst the three locations was in Belo Horizonte (65.2%), which is lower than the average reported in other studies of the informed consent process in developing countries [40–42], in developed countries [43], and in both types of countries [21]. Regardless of the setting in which the research is conducted, a lack of understanding of the information about the research impairs the quality of the individuals’ decision to participate [44].

The inability to describe the risks and possible adverse effects of participation in a particular clinical trial, observed mostly in Americaninhas and in Washington, DC, has been seen in other studies of the informed consent process, such as in a breast cancer clinical trial conducted in Japan [45]. In that study, the scores achieved by participants were acceptable in terms of a broad understanding of the informed consent document, but were low for particular items such as the experimental nature of the study, potential risks, benefits, and compensation.

The limited understanding of information contained in the informed consent form may influence the voluntariness of an individual’s decision to participate [8]. In this sense, the results of the questionnaire related to knowledge about the clinical trial discussed above suggest that the informed consent obtained in Americaninhas may have been relatively compromised in relation to the voluntary decision to participate, in comparison to other places. Other aspects related to the willingness of volunteers from Brazil to participate in the study consist of potential indirect benefits associated with participation in the clinical research, such as learning about the disease and receiving a medical examination as part of screening. While such care is provided free of charge by the universal Brazilian public system of health in Americaninhas, access to medical care is hampered due to a shortage of medical professionals in the region and to excess demand, conditions that may have had a direct influence on willingness to participate in the study.

Aspects related to the participants’ attitudes also influence the voluntariness and the quality of the informed consent, such as fear of participation, trust in the study team, the expression of doubt, and the underlying intentions and motivations for participating. Regarding the voluntariness of the decision to participate in the clinical trials that were the subject of the current research, most participants at all three sites reported not being afraid of participating and having trust in the investigators who were responsible for the clinical trials, an important finding that contributed to the quality of the informed consent in both countries. Jenkins and Fallowfield remark that fear and dissatisfaction with certain research procedures were cited as reasons for people declining participation in cancer research studies [46]. Despite this, a study aimed at analyzing attitudes toward participation in clinical research in a developing country demonstrated that the majority of respondents reported that they would not like to entrust decisions about their health to physicians. Only a small proportion of participants, particularly those without any formal education, would leave the process of making health decisions in the hands of physicians [47].

The study participants from Americaninhas, unlike those from Belo Horizonte or Washington, DC, had very few doubts or concerns about the clinical research in which they were participating. This might be explained by a reduced capacity to ask questions during the informed consent process due to lower levels of education [48]. In addition, we found that the popular image of the physician overlapped with the image of the researcher in this community, something previously observed in this region [49]. This situation might intimidate study volunteers, making them less likely to ask critical questions of the researchers [40]. It has previously been shown that clinical trial participants with higher levels of education are more inclined to discuss their potential participation in the study with the research team [35]. In contrast, in Americaninhas the belief that participation in a clinical trial may improve their health status, may lead them to be less critical of the research aspects of the study [50].

Regarding the desire to enroll in the clinical trial, we found that most participants actively wished to participate; however, paradoxically, in Americaninhas, half merely “tolerated” participation. This contradiction might be attributed to two factors: a difficulty in understanding the question, despite the care taken during questionnaire preparation and implementation; or, a limited voluntariness in the decision-making process, resulting from a lack of understanding of the information about the trial, third-party influences, or the level of trust in the researchers [8]. Another aspect that may be associated with limited voluntariness and that may help to explain this contradiction is the notion that participation in the clinical trial might improve one’s health. Especially in developing countries, the level of trust in the investigators conducting a clinical trial might influence a reluctant volunteer during the consent process due to their degree of authority in such an environment; a potential research participant might feel that participation cannot be refused to such an individual [51]. For example, a review by Mandava *et al* revealed that participants from developing countries are less likely to refuse participation in research and are more likely to worry about the consequences of their refusal to participate [11].

Regarding the motivation for participating in the clinical trial conducted in Americaninhas, most individuals attributed it to a personal decision taken of their own free will. Such motivation may be due to the endemic level of hookworm infection in the study area; that is, proximity to the disease being studied might lead the volunteer to associate participation in the clinical trial with the prospect of improving their health. In contrast, in both Belo Horizonte and in Washington, DC, the participants reported that their participation was motivated by the possibility of helping other people, perhaps driven by their reduced exposure to the disease for which the vaccine is being developed. Specifically in the case of the United States, volunteers named the financial benefit deriving from their participation as a major factor in enrolling in the research. Receiving a financial incentive for participation in clinical trials was reported as a reason for enrolling in another healthy volunteer study conducted in New Haven, Connecticut, in which 58% of the respondents reported it as the primary motivation for participating [52]. The same study identified a positive correlation between financial interest and a greater understanding of the informed consent document, an aspect that could not be evaluated in the current study given its dissimilar objectives and methodology and the fact that monetary compensation for clinical trial participation is not permitted in Brazil.

The findings of our study are supported by the fact that we utilized a set of strategies employed to ensure method reliability, internal data validity, and minimization of bias. First, the questionnaires used open-ended questions to assess the participants’ knowledge of information contained in the informed consent document. This type of tool is able to measure more accurately the actual knowledge of a topic, and avoids overestimation of responses or influencing responses by presenting pre-determined options [26, 28]. We also sought to ensure that knowledge of the information conveyed in the informed consent form derived merely from the reading of the document and was not influenced by the experience of participating in the trial. This was achieved by administering the questionnaires after the consent procedure but before the first study vaccination. In most cases the questionnaire was administered on the first day of vaccination although in a handful of cases it was administered on the same day as consent was obtained; it is possible that differences in time between signing the consent form and completing the questionnaire may have affected the observed results, however the number of participants who completed the questionnaire immediately was too small to make valid comparisons. This is a potential limitation of our research, as is the fact that the informed consent forms were not identical between the studies conducted in Brazil and in the United States due to slight differences in study design, primarily the use of different adjuvants in the two trials. However, this is unlikely to have significantly impacted our results since the major differences observed were between participants in Americaninhas and those in Belo Horizonte and Washington, DC, rather than between the Brazilian and American studies.

The validity of the data was also ensured by standardizing the questions and how the responses were recorded, thereby increasing reliability and permitting more robust statistical analysis of the data [53]. Standardization was essential to compare the participants’ knowledge, especially since the questionnaires were administered in three diverse study locations. In order to ensure standardization of the process, interviewers received training prior to the application of the questionnaire.

The questionnaire used in this research addressed all of the necessary aspects of an ethically sound informed consent, in contrast to the study by Ellis et al in which only seven themes were evaluated [21]. This same study pioneered the comparison between levels of quality of informed consent in developed (USA) and developing (Mali) countries. However, the investigators used a questionnaire with a primarily educational purpose that did not take into consideration the rigor of scientific research. In order to build upon the findings of this study, the questionnaire design used in our research was based on other tools validated for this purpose and on the researchers’ experience developing and administering similar surveys [7–8, 27, 54].

Despite the fact that international ethical guidelines and literature on the subject of informed consent call for additional measures to protect the rights of research participants in developing countries, the findings of this study suggest that the characteristics of participants at each specific study site need to be considered, regardless of the country in which they are located [1, 26]. This observation was emphasized by Lobato [55], who demonstrated that certain characteristics of study participants may be negatively associated with the quality of the informed consent that is obtained, particularly those associated with extrinsic or intrinsic vulnerability [16].

## Conclusions

The study described herein provides the first empirical comparative analysis of the quality of the informed consent process of participants in a clinical trial in a developed country with participants from Brazil. In addition, it compared the quality of the informed consent process in different Brazilian contexts. Regarding the methodology, this is the first study to investigate the theme using clinical trials of similar design and testing the same investigational products, and that measured results through a standardized questionnaire designed specifically for that purpose. From this perspective, it may contribute especially to building a body of knowledge about the quality of informed consent worldwide.

As described above, potential limitations of this study include the lack of validation of the questionnaire prior to its use, as well as the different methods of its implementation in both countries. Furthermore, it is understood that a larger sample of participants could give a different result is that the same sample used could produce other results in different population groups and ages or in other countries, or in different locations within Brazil and the US given local differences.

Despite this, based on our results, we conclude that the use of the terms “developed” and “developing” to describe countries is a reductionist exercise to define participants as vulnerable, whereas a rigorous consideration of the specific characteristics of each group of individuals recruited as participants in a clinical trial is necessary. These findings demonstrate also the need for educational interventions directed at clinical trial participants, both in developing and developed countries, in order to improve understanding of the informed consent document.

## Supporting Information

S1 Checklist STROBE Checklist. (PDF)

S1 ICF Informed consent form used in Belo Horizonte, Brazil, for clinical trial SVI-10-01 (English translation). (PDF)

S2 ICF Informed consent form used in Americaninhas, Brazil, for clinical trial SVI-10-01 (English translation). (PDF)

S3 ICF Informed consent form used in Washington, DC, for clinical trial SVI-GST-03. (PDF)
