# Supplementary material for: A Comparison of the Quality of Informed Consent for Clinical Trials of an Experimental Hookworm Vaccine Conducted in Developed and Developing Countries
Source: PLoS Negl Trop Dis. 2017 Jan 23;11(1):e0005327. doi: 10.1371/journal.pntd.0005327 (PMC5289607; doi:10.1371/journal.pntd.0005327)
Supplement: S2 ICF — (PDF) [file pntd.0005327.s003.pdf]

INFORMED CONSENT FORM FOR A NEW RESEARCH PROJECT – STUDY PART II

Title of Research Project: Phase 1 Study of the Safety and Immunogenicity of *Na*-GST-1/Alhydrogel® with or without GLA-AF in Brazilian Adults

INTRODUCTION

We are asking you to participate in a research study for a new hookworm vaccine. This consent form gives you information about the study that will be discussed with you. Once you understand the study, and if you agree to join, we will ask you to sign or put your fingerprint on this form. Two copies of this document will be signed. We will give you one copy to keep and the other one will be stored by the investigator, so that there will be two signed copies.

First, we want you to know that:

- Your participation in this research study is optional.
- You do not have to take part in this study, or you can decide later to leave this study if you change your mind.
- If you decide to leave the study, nothing that you have received will be taken away from you.

Before you decide to be in this research study, please take as much time as you need to ask any questions and discuss this study with people on the research team, or with family, friends, or people from your community. You may also ask questions at any time after joining the study.

WHO IS SPONSORING THIS STUDY?

This research study is being done by Centro de Pesquisas René Rachou FIOCRUZ in Belo Horizonte, and is being funded by the Sabin Vaccine Institute, located in the United States. The people responsible for the study are Dr. David Diemert, a physician at the Sabin Vaccine Institute in the United States and at FIOCRUZ in Belo Horizonte, and Drs. Vanderson Valente, Janaina Freire, Frederico Talles, and Geraldo Castro, all from FIOCRUZ.

WHAT IS A HOOKWORM VACCINE?

Scientists at FIOCRUZ and the Sabin Vaccine Institute are doing research to make a vaccine against hookworm. This new vaccine against hookworm is a protein – *Na*-GST-1 – that comes from a small part of the hookworm parasite. This vaccine is being tested and is not yet approved for use in Brazil, the United States, or anywhere else. At this time, there is no other vaccine for hookworm. We hope that this study will give us information that can be used to develop a hookworm vaccine that will help stop people from getting sick from this worm.

WHY ARE WE ASKING YOU TO TAKE PART IN THIS STUDY?

We are asking you to be in this study because you live in an area of Brazil where many people get worms. Hookworm is one of many types of worms that are found in the area that you live. Anyone can get hookworm if their skin touches dirt or water that is contaminated with hookworm larvae. Larvae in the dirt or water come from the feces of people who are already infected with hookworm. Sometimes you can feel sick from the worms and other times you may not know that you have them. When you have hookworms you may feel tired and weak.

We are asking you to be in this study because the researchers want to test a new vaccine against hookworm to see if the vaccine is safe, before testing it in more people or in children. Only adults between the ages of 18 and 45 years can be in this study. The vaccine we will test in this study is only against hookworm and it will not have any effect on other kinds of worms.

WHY ARE WE DOING THIS STUDY?

In this study, we want to find out:

- If the new hookworm vaccine is safe when given to adults
- How your body reacts to the vaccine

If the vaccine is safe when given to you and the other participants in this study, the researchers will test it in children who live in the northeast region of Minas Gerais.

HOW LONG WILL THIS STUDY LAST?

Your participation in this study will last for 16 months. If you think that you are going to move away during this time, you will not be able to join this study because you need to be here for the whole study.

WHAT DO I HAVE TO DO IN THIS STUDY?

If you agree to be in this study, the first visit will be a screening visit. We will ask you some questions about your health. We will also ask you to answer a questionnaire about your understanding of the study and your reasons for participating. You do not have to answer this questionnaire if you do not want to and you can still participate in the study if you don't answer the questionnaire.

One of the research medical doctors will examine you to see if you are in good health. A small amount of blood (around 30 mL) will be taken from your arm to do some lab tests. Your blood will be tested to check the function of your blood, your liver and your kidney, and to see if you are infected with viral hepatitis or HIV. You will give us a stool sample so we can check if you have a worm infection. The medical doctor will have to wait for your test results to come back from the lab before we will know if you can be in the study. If you are a woman, we will ask you to give us some of your urine to see if you are pregnant. Pregnant women and women who are breastfeeding cannot be in this study.

If your blood tests show that you have an illness, or if you are not in good health, you will not be able to be in this study. The researchers will discuss this with you and will make sure that you are followed up at the health post in your community. If you are infected with worms, you can still be in the study, but only if you agree to be treated with medication for the infection before you get any injections.

If the screening tests and the medical exam show that you can take part in this study and if you still want to be in the study, we will ask you to come to the study clinic to start the study. It is possible that we will not choose you to be in the study even though we ask you to come to the clinic. If this happens, we will ask you if you want to receive the hepatitis B vaccine instead of being in the study. If you are chosen to be in the study, you will receive a total of three injections of the new hookworm vaccine over a period of 4 months in one of three different doses of the vaccine: low, medium, or high, or the hepatitis B vaccine (the same one that is given by the government of Brazil). We are trying to find the dose that is the safest and that gives the best chance of preventing hookworm disease. In addition, we are trying to see IF a new immunostimulant called GLA-AF can increase the strength of the vaccine.

The injections will be given in your upper arm muscle using a needle. If you are a woman, your urine will be tested before every injection to make sure you are not pregnant. If you are pregnant, you will not be allowed to receive any other injections, but we still will still follow you at the study clinic – without any cost to you – so that we can check if you and your baby stay healthy. We will follow you at least until the end of your pregnancy, during delivery, and if necessary, after delivery for as long a period as is necessary.

In this part of the study in the region of Americaninhas, 30 people in the study will get the new hookworm vaccine, 30 people will get the new hookworm vaccine mixed with the immunostimulant GLA-AF, and 6 will get the hepatitis B vaccine. You, the doctors and other members of the study team will not know which vaccine you get. The decision will be made by chance, like when you toss a coin and you don't know what side it will land on. Everyone will get three injections. The second injection will be given 2 months after the first injection, and the last shot will be given 2 months after the second. You will get the same vaccine for each of the three shots. At the end of the study, we will tell you which vaccine you got. The hepatitis B vaccine will protect you against hepatitis B

infection, but it will not stop you from getting hookworm.

Every time that you get an injection, you will have to stay in the study clinic for at least two hours to make sure you do not start feeling bad. We will also examine you in the clinic 1, 3, 7, and 14 days after each injection. At these visits we will ask you how you are feeling and examine you. We will also take some of your blood (around 50 mL) on the days that you get the injections, and 14 and 28 days after each injection.

We will ask you to return to the clinic for follow-up 3, 6, 9 and 12 months following your final injection. At each follow-up visit we will take some of your blood (up to 50 mL). We will ask you to give us a stool sample to check for worm infections 1 month after the last injection, and at the end of the study. If you are infected with worms at the end of the study, we will give you medicine if you want it. There will be no charge for this medicine and we will give it to you for free. You can ask to one of the research medical doctors to explain to you the risks and benefits of these medicines.

During this study, we will take blood from you 13 times, and the total amount of blood will be around 550 ml. Your blood will be tested to check the function of your blood, your liver and your kidney, and to measure how your body responds to the vaccine. More blood tests might be needed if you get sick. Some of your blood will be stored at FIOCRUZ in Belo Horizonte initially for 5 years, and possibly for longer. This material may be used to do more tests on your blood if you become sick, or in future research using tests like those described in this document, or different tests. Storage for longer than 5 years or use of your blood in conducting other research will only be done after getting approval from the ethics committee at FIOCRUZ.

CAN I GET HURT IF I AM IN THIS STUDY?

This is the first time that the new *Na*-GST-1 hookworm vaccine will be given to people. We do not know if the new hookworm vaccine will protect you from hookworm infection, and there is a chance that it will not work at all.

When you get the new hookworm vaccine (with or without the additional immunostimulant) or the hepatitis B vaccine, you can expect some soreness and possibly some redness where the injection was given. You could also feel pain and itching and have purple or red spots on your skin. Some people feel a little sick for a few days after getting an injection. You could feel periods of hot and cold, headache, stomach ache, and sore muscles and joints. Some of this can happen several weeks after you receive the injection.

With any vaccine, there is a very small chance that a serious and sometimes deadly allergic reaction might happen within the first hour after the injection is given. These feelings can start with your tongue swelling, feeling lightheaded or dizzy, or having a hard time breathing. Because of this you will be watched carefully for 2 hours after each injection. There may be other bad reactions that we don't know about yet. If we do find out about a new reaction to the hookworm vaccine or the hepatitis B vaccine, we will tell you. The research doctors and nurses will take good care of you if you have any problems with the injections. If you have a bad reaction to an injection you will not get any more injections.

The new *Na*-GST-1 hookworm vaccine has never been tested in pregnant women and might be dangerous for the baby. If you are a woman, to be in this study you will have to use two methods of birth control, such as birth control pills and condoms, until 1 month after the last injection. We can help you pick the best methods, or we can send you to the health outpost to discuss the best birth control methods. We will give you the birth control that you want to use, for free.

The blood tests may cause a little pain when the needle is put in the arm. Later the arm may have some bruising (purple spots) or bleeding where the needle went in, and rarely can get infected. Sometimes drawing blood causes people to feel lightheaded and even faint.

WHAT IF I GET HURT WHILE IN THIS STUDY?

If you get hurt or sick during the study, you should tell someone on the study team immediately, and we will give you medical care for free. The research doctors and nurses will make sure you get good treatment for any pain or injuries that are caused from taking part in this study. You will also receive comprehensive free care that is guaranteed by the sponsor of the study, in case of injury due to your participation, without any limitation or condition and the legal right to receive compensation.

ARE THERE ANY BENEFITS TO BEING IN THIS STUDY?

You may not directly benefit from being in this study. However, during the study you may learn about worms and how to avoid them. Also, you will receive medical care during the study at the study clinic in Americaninhas. At the end of the study, we will tell you what vaccine you got. If you got the new hookworm vaccine, you can come to the clinic after the end of the study to get the hepatitis B vaccine if you want it. If you got the hepatitis B vaccine during the study, we will test some of your blood that we will collect at the end of the study to see if the vaccine worked and if you are protected against hepatitis B infection. If you are not protected, we will give you a second hepatitis B vaccine if you want it, at no cost to you. The hepatitis B vaccine will be given to you in three injections over a period of 6 months.

DO I GET ANYTHING FOR BEING IN THIS STUDY?

You will not receive any money if you join this study. If you have to pay to come to the clinic for the study visits, we will pay for this transportation. Also, we will give you a meal at the clinic on the days that you get your injections.

DO I HAVE ANY OTHER OPTIONS BESIDES BEING IN THIS STUDY?

You do not have to be in this study, and no one will be mad at you if you decide you do not want to do it. Medical care and health exams are available at the health post in your community. If you decide not to be in this study it will not affect your current or future medical care at the health post or at any other place.

WHAT ARE SOME OF THE REASONS I MIGHT NOT BE ABLE TO FINISH THE STUDY?

The research doctors may decide that it is not good for you to stay in the study because it might be dangerous to your health. Also, the study team might stop the whole study from finishing.

HOW WILL MY INFORMATION BE KEPT PRIVATE?

Information about you in this study will be kept in a secure place. People who need to make sure that the research is done well may look at your records. This might include the study staff, the sponsor of the study (the Sabin Vaccine Institute) or people they designate, the ethics committees of FIOCRUZ in Belo Horizonte, the Brazilian federal government, The George Washington University, and the Food and Drug Administration of the United States federal government. All of these people will keep your information private. We will not give any information that identifies you to anyone who is not working on the study.

WHO CAN I TALK TO IF I HAVE QUESTIONS?

For questions about your rights as a volunteer in research:
Research Ethics Committee: Dr. João Carlos Pinto Dias
Fundação Oswaldo Cruz (FIOCRUZ), Centro de Pesquisas René Rachou
Avenida Augusto de Lima 1715, Barro Preto, Belo Horizonte, Tel: (31) 3292-7825

For questions about the study:
Principal Investigator: Dr. David Diemert
Fundação Oswaldo Cruz (FIOCRUZ) - Centro de Pesquisas René Rachou
Avenida Augusto de Lima 1715, Barro Preto, Belo Horizonte, MG, Brasil – Tel: (31) 3349-7715

For questions about the study or if you want to report an illness:
Field Manager: Renata Caldeira Diniz
Fundação Oswaldo Cruz (FIOCRUZ) - Centro de Pesquisas René Rachou

Avenida Augusto de Lima 1715, Barro Preto, Belo Horizonte, MG, Brasil – Tel: (31) 3349-7715 or (33) 3532-7014 (available 24 hours)

I have read, discussed, and understood this informed consent form. My questions were answered. I freely consent to participate in this study.

……………………………………………………………………………………………

Volunteer's Name (printed letters)

……………………………………………………………………………………………..

Volunteer's Signature/Thumbprint

Date: |__|__|/|__|__|__|__|/|__|__|__|__|

Person Obtaining Consent:

……………………………………………………………………………………

Name (stamp or printed letters)

……………………………………………………………………………………

Signature

Date: |__|__|/|__|__|__|__|/|__|__|__|__|

Can the volunteer read? |__| Yes |__| No *(requires presence and signature of a witness)*

Witness (if volunteer is not able to read and understand the Informed Consent Form):

I affirm that the Informed Consent Form has been read to the volunteer and he/she understands the study and I have witnessed the volunteer's consent to participate in the study.

…………………………………………….. ……………………………………………….…

Name (printed letters) *Signature*

Date: |__|__|/|__|__|__|__|/|__|__|__|__|

Principal Investigator or Designee: Name*:* …………………………………………………….…

(stamp or printed letters)

Signature: ……………………………………………………………………………………………..

Date: |__|__|/|__|__|__|__|/|__|__|__|__|
