# Supplementary material for: A Comparison of the Quality of Informed Consent for Clinical Trials of an Experimental Hookworm Vaccine Conducted in Developed and Developing Countries
Source: PLoS Negl Trop Dis. 2017 Jan 23;11(1):e0005327. doi: 10.1371/journal.pntd.0005327 (PMC5289607; doi:10.1371/journal.pntd.0005327)
Supplement: S3 ICF — (PDF) [file pntd.0005327.s004.pdf]

Study Title: Phase 1 Study of the Safety and Immunogenicity of *Na*-GST-1/Alhydrogel®, With or Without a CpG ODN Adjuvant, in Healthy Adults

Principal Investigator: David Diemert, MD

You have been asked to participate in a new research study. Before you decide whether you want to participate in the study, you have a right to the following information:

1. The nature and purpose of the study.
2. The vaccines that will be used in the study.
3. The procedures that will be performed in the study.
4. Risks and discomforts that can reasonably be expected from the study.
5. Benefits that can reasonably be expected from the study.
6. Alternatives to participating in the study.
7. Availability of medical treatment should you become injured as a result of the study.
8. The opportunity to ask questions about the study.
9. The right to stop participating in the study at any time without any penalty.
10. A copy of this signed and dated written consent form for you to keep.

INFORMED CONSENT FOR A NEW RESEARCH STUDY

Study Title: Phase 1 Study of the Safety and Immunogenicity of *Na*-GST-1/Alhydrogel®, With or Without a CpG ODN Adjuvant, in Healthy Adults

Principal Investigator: David Diemert, MD

Study Sites: George Washington Medical Faculty Associates and George Washington University, Department of Microbiology, Immunology and Tropical Medicine

24-Hour Telephone Number: (202) 270-2393

We are inviting you to take part in a research study. This research study is sponsored and being paid for by the Sabin Vaccine Institute (Washington, DC). The person in charge of this study will be Dr. David Diemert, MD, of the George Washington University and the Sabin Vaccine Institute. Please take as much time as you need to read this consent form. You may want to talk about it with your family, your friends, or your personal doctor. You may find parts of this form hard to understand. If you do, please ask questions. Participation in this research study is voluntary. If you choose to join the study, you will be asked to sign this form. We will give you a copy of the signed form to keep.

WHY ARE WE DOING THIS STUDY?

We are doing this study to find out if a new experimental hookworm vaccine is safe when given to healthy adults. A vaccine is something that is used to prevent an infection or disease. Researchers from the Sabin Vaccine Institute and the George Washington University are trying to make a new vaccine for hookworm because right now there is NO vaccine for this important disease. We want to test this new hookworm vaccine when it is mixed with a new adjuvant that is called “CpG”. An adjuvant is something that is added to a vaccine to make it work better. We hope to learn if your body responds to the vaccine and adjuvant by making antibodies. Antibodies (germ fighters) are proteins that your body makes that protect you from infections.

The Sabin Vaccine Institute is making the new hookworm vaccine to help protect people from hookworm. Hookworm is a parasitic worm that can live for several years in the intestine (gut) of humans. Hookworms feed on the blood of the people they infect, which can cause anemia (low blood counts) and other illness. People get infected with hookworm when their skin comes into contact with tiny larvae (baby worms) that live in soil that has been contaminated with human fecal waste that has not been properly treated. Hookworm infection does not happen in the United States, but it is very common in poor parts of South America, Africa and Asia. Over 500 million people in the world are living with hookworm infection right now. We hope that this study will give us information that can be used to develop a hookworm vaccine that will help stop people from getting sick from this worm.

This new vaccine is made from a protein called *Na*-GST-1 that comes from a small part of the hookworm. You cannot get hookworm disease from the vaccine. The *Na*-GST-1 protein has been put into vials with an adjuvant called “aluminum hydroxide” that is included in many

licensed vaccines such as the hepatitis B vaccine and the tetanus vaccine. We want to test the *Na*-GST-1 vaccine when it is mixed together with a second adjuvant called "CpG 10104". CpG is an experimental adjuvant made of a small length of synthetic DNA. We want to see if this new CpG adjuvant will boost your body's response to the vaccine.

The hookworm vaccine is not yet approved for use in the United States, or anywhere else. The vaccine has been tested in 40 healthy adults in the US and in 96 healthy adults in Brazil using other adjuvants. This is the first time this specific CpG will be used in humans.

You can take part in this study if you are a **HEALTHY MAN OR WOMAN** between the ages of 18 and 50 years and live in the Washington, DC metropolitan area. Up to 24 people will take part in this study.

WHAT IS INVOLVED IN THE STUDY?

We want to compare the immune (infection fighting) responses of participants who receive only the hookworm vaccine by itself and participants who receive the hookworm vaccine plus the new CpG adjuvant. Different participants will receive different amounts of the vaccine or the vaccine mixed with the new CpG adjuvant. Two different amounts of vaccine (30 and 100 micrograms) with and without the adjuvant (500 micrograms) will be used in the study. This will allow us to see how safe different amounts of the vaccine are with the adjuvant. We can also see if there is a difference in the amount of antibodies (germ fighters) that your body makes to the different combinations of the vaccine and adjuvant.

To answer these questions, we will divide participants into 2 groups of 12 people each. In each group, some participants will get the vaccine *plus* the CpG adjuvant, while some will get the vaccine *without* the CpG. Whether you get the vaccine *plus* the CpG or *without* the CpG will be determined by chance, kind of like flipping a coin. Neither you nor the study team in the clinic will know if you got the vaccine *plus* the CpG or the vaccine *without* the CpG. We will tell you which vaccine you got at the end of the study.

We will assign you to a group based on the order that you join the study, kind of like being in line at a store checkout. If the first group fills up before you are registered in the study you may be asked to join the second group. You cannot ask to be in a specific group. The vaccine and new CpG adjuvant will be given together in the same injection.

- 12 people will be in **Group 1:** 8 participants will get the low amount of vaccine *plus* the CpG adjuvant, 4 participants will get high amount of vaccine *without* the CpG.
- 12 people will be in **Group 2**: 8 participants will get the high amount of vaccine *plus* the CpG adjuvant, 4 participants will get the high amount of vaccine *without* the CpG.

Your first visit (this visit) will be a screening visit. If you don't want to do everything in one visit, you can come back later to finish the screening. During screening, the following things will happen:

- You will read this informed consent form. After this you will decide if you want to participate in the study or not.
- If you want to participate, you will sign this informed consent form.
- Next, we will check if you can join the study. You will be asked questions about your health and have a physical examination.
- We will take a blood sample (about 2 tablespoons) from your arm that will be tested for the following:
  - A complete blood count
  - Tests of your kidney and liver function
  - Tests for HIV, Hepatitis B, and Hepatitis C
  - Tests for autoimmune diseases, such as lupus or rheumatoid arthritis (autoimmune diseases are diseases where the body makes an immune response against itself)
- You will give a urine sample that will be tested to see if there is blood or protein in it. If you are a woman, your urine will also be tested to see if you are pregnant.

We will have to wait for your blood test results to come back from the lab before we will know if you can be in the study. If your examination or blood tests show that you have an illness, or if you are not in good health, you cannot be in this study. You must have a negative pregnancy test (if you are a woman) and negative tests for HIV, Hepatitis B, Hepatitis C and autoimmune diseases to be in this study. The study staff will tell you your results and refer you for follow-up care if needed.

If you test positive for HIV or Hepatitis B or C virus, we are required by law to notify the DC Department of Health. We will have to tell the Department of Health your name and other personal identifying information, but they will keep this information secret.

If your screening tests show that you can participate in the study, and you decide that you want to participate, you will be in the study for 16 months. During this time, you will receive three vaccinations of the hookworm vaccine given as an injection (shot) in your upper arm muscle (deltoid) using a needle and a syringe. After the first injection is given, the second injection will be given 2 months later, and the third injection 4 months after the first one. You will get the same vaccine at each injection. You will visit the clinic 18 times (including the screening visit). 14 visits will last for less than 1 hour. The screening visit will last about an hour and the 3 vaccination visits will last about 1½ hours. You will also be contacted by telephone twice. Each call will last less than 30 minutes. The total amount of time is about 16 hours. There will be a visit at 3, 7, 14 and 28 days after each injection. Then we will see you 3 and 6 months after the last injection, and call you by telephone 9 and 12 months after the last injection.

We will take some blood from you 15 times during the study. The amount of blood taken each time will be between 1 to 3½ tablespoons (15 mL to 50 mL). The total amount of blood we will take during the whole study will be about 620 mL (about 2½ cups). During the study, your blood

will be tested to check blood cell counts and your kidney and liver function to be sure that you stay healthy. Some blood will also be used to see if your body is making antibodies (germ fighters) and other things that help the body fight infections. If you are a woman, we will also take urine samples on each vaccination day to see if you are pregnant.

- **Screening Visit:** described above
- **Visit 1: Vaccination #1**
  - We will take a history of symptoms since your last visit
  - You will have a brief physical exam
  - You will give a blood sample (about 3½ tablespoons)
  - If you are female, you will give a urine sample
  - You will be given an injection of vaccine or vaccine with adjuvant
  - You must stay in the clinic for 1 hour after your injection to see if you have any reaction
- **Visit 2: 3 days after 1st injection**
  - We will take a history of symptoms since your last visit
  - You will have a brief physical exam
- **Visit 3: 7 days after 1st injection**
  - We will take a history of symptoms since your last visit
  - You will have a brief physical exam
  - You will give a blood sample (about 2 tablespoons)
- **Visits 4 and 5: 14 and 28 days after 1st injection**
  - We will take a history of symptoms since your last visit
  - You will have a brief physical exam
  - You will give a blood sample (about 3 to 3½ tablespoons)
- **Visit 6: Vaccination #2**
  - We will take a history of symptoms since your last visit
  - You will have a brief physical exam
  - You will give a blood sample (about 3½ tablespoons)
  - If you are female, you will give a urine sample
  - You will be given an injection of vaccine or vaccine with adjuvant
  - You must stay in the clinic for 1 hour after your injection to see if you have any reaction
- **Visit 7: 3 days after 2nd injection**
  - We will take a history of symptoms since your last visit
  - You will have a brief physical exam
- **Visit 8: 7 days after 2nd injection**
  - We will take a history of symptoms since your last visit
  - You will have a brief physical exam
  - You will give a blood sample (about 2 tablespoons)

- **Visits 9 and 10: 14 and 28 days after 2nd injection**
  - We will take a history of symptoms since your last visit
  - You will have a brief physical exam
  - You will give a blood sample (about 3 to 3½ tablespoons)
- **Visit 11: Vaccination #3**
  - We will take a history of symptoms since your last visit
  - You will have a brief physical exam
  - You will give a blood sample (about 3½ tablespoons)
  - If you are female, you will give a urine sample
  - You will be given an injection of vaccine or vaccine with adjuvant
  - You must stay in the clinic for 1 hour after your injection to see if you have any reaction
- **Visits 12: 3 days after 3rd injection**
  - We will take a history of symptoms since your last visit
  - You will have a brief physical exam
- **Visit 13: 7 days after 3rd injection**
  - We will take a history of symptoms since your last visit
  - You will have a brief physical exam
  - You will give a blood sample (about 2 tablespoons)
- **Visits 14 and 15: 14 and 28 days after 3rd injection**
  - We will take a history of symptoms since your last visit
  - You will have a brief physical exam
  - You will give a blood sample (about 3 to 3 ½ tablespoons)
- **Visit 16: 3 months after 3rd injection**
  - We will take a history of symptoms since your last visit
  - You will have a brief physical exam
  - You will give a blood sample (about 3 tablespoons)
- **Visit 17: 6 months after 3rd injection**
  - We will take a history of symptoms since your last visit
  - You will have a brief physical exam
  - You will give a blood sample (about 3 tablespoons)
- **Telephone call #1: 9 months after 3rd injection**
  - We will call you and take a history of symptoms since your last visit
- **Telephone call #2: 12 months after 3rd injection**
  - We will call you and take a history of symptoms since your last visit

We will give you a thermometer and a diary card and ask you to record your temperature and symptoms daily for the first 7 days after each of the three injections.

Information about Samples Collected as Part of This Research:

If you do not want to have blood collected you will not be able to participate in the study.

Extra blood may be stored for future research. If you do not want your samples stored for future research you can still participate in the study. At the end of this consent form, you will be asked to decide if we can keep your samples for future testing.

WHAT ABOUT PREGNANCY?

We do not know whether the vaccine or the new CpG adjuvant might hurt an unborn baby. If you are pregnant, you cannot participate in this study. If you are a woman who could become pregnant, you must have a urine pregnancy test to make sure you are not pregnant. We will do a pregnancy test on each day that you will be vaccinated. If you are sexually active or plan to be during the study, you must use effective birth control during the study until at least 1 month after the last injection. These are some birth control measures that you can use:

- Hormonal contraceptives (such as birth control pills, implants, or injections)
- Barrier methods (such as a condom or diaphragm) used with a spermicide
- An intrauterine device (IUD)
- Surgical sterilization (hysterectomy or tubal ligation)

If you become pregnant during the study, you will not get any more injections of the vaccine or adjuvant. However, we will monitor you closely until the end of the pregnancy for your safety the safety of your child.

If you are breastfeeding and do not want to stop, you cannot take part in this study.

WHAT ARE THE POSSIBLE RISKS AND DISCOMFORTS?

The risks and discomforts associated with participating in this study are mainly those associated with the experimental vaccine and new CpG adjuvant. When you get the new hookworm vaccine, with or without the CpG adjuvant, you can expect some soreness and possibly some redness where the injection was given. You may feel discomfort lifting your arm in which the injection was given. You may also feel pain and itching and have purple or red spots on your skin. Some people feel a little sick for a few days after getting an injection. You could feel periods of hot and cold, headache, stomach ache, and sore muscles and joints. Some of this can happen several weeks after you receive the injection. With any vaccine, there is a very small chance that a serious and sometimes deadly allergic reaction might happen within the first hour after the injection is given. These feelings can start with your tongue swelling, feeling lightheaded or dizzy, or having difficulty breathing. Because of this you will be watched carefully for at least 1 hour after each injection. There may be other bad reactions that we do not know about yet. If we do find out about a new reaction, we will tell you. If you have a bad reaction to an injection you will not get any more injections.

CpG has been used in other types of vaccines, but this is the first time this specific CpG will be given to people. CpG may potentially over-activate the immune system and cause the body to make an immune response against itself. The result of this can be a type of disease called an

autoimmune disease. Examples of autoimmune diseases include lupus, rheumatoid arthritis, Sjogren's syndrome, and thyroid inflammation. You will be checked for any evidence of an autoimmune disease before you begin the study and throughout the study.

The possible risk of developing an autoimmune disorder is low. Among 4425 volunteers who were vaccinated with a product that has some similarities to the adjuvant being tested, 3 developed new autoimmune disorders (Wegener's granulomatosis, Tolosa-Hunt syndrome, and Guillain-Barré syndrome). In this study, among the 1420 individuals who were vaccinated with a licensed vaccine that does not contain CpG, 1 developed an autoimmune disorder (ANCA-positive vasculitis). Therefore, the rates were about the same in the two groups.

Another risk of CpG is developing an immune response against DNA. You will be checked frequently for immune responses to DNA during the study. You may experience changes in the counts of your white blood cells (the blood cells that help fight infection) and in the platelets (the cells that help with blood clotting). Your blood count will be frequently checked during the study.

You are also likely to experience some discomfort from having blood drawn from you. You may feel some pain and bruise at the site where we take the blood from, or have some dizziness. Rarely, infection occurs where the needle enters the skin.

WILL YOUR INFORMATION BE KEPT PRIVATE?

We will keep your information private to the extent possible by law. Federal laws require that researchers and health care providers protect the privacy of information that identifies you and relates to your past, present and future physical and mental health or conditions, or the provision of health care. If you agree to take part in this study, protected health information will be used and shared with others for the purposes of the study. By signing this form, you are allowing the people and groups that are listed in the next paragraphs to use your health information for this research study. Your information will only be used or shared as explained in this informed consent form.

The use and release of protected health information is for the purpose of collecting information for this study. Protected health information to be shared includes your name, address, telephone number, birth date, diagnosis, medical history, and test results.

The study doctors and other members of the study team may obtain your individual health information from hospitals, clinics, health care providers, and health plans that provide care to you during the study. By signing this form, you allow the use, sharing, copying, and release of your protected health information in connection with this study by:

- The members of the research team;
- Other healthcare providers such as labs which are part of the study;
- A safety monitoring committee that will be monitoring your safety during the study;
- Institutional officials who are responsible for compliance;
- Representatives and agents of the sponsor of the study;
- Representatives of federal regulatory agencies such as the U.S. Food and Drug Administration (FDA).

All of the tests we will do in this study will be done only because you are in this study. The results of these tests will not be sent to your primary doctor or included in your medical record. Once your health information has been given to others outside of the study team the information may no longer be covered by the federal regulation that protects privacy of health information.

If you don't sign this form or if you cancel your permission later, it will not affect your health care treatment outside of the study, payment for healthcare from a health plan, or ability to get health plan benefits. However, if you do not give permission to use your protected health information you will not be able to take part in this study because your protected health information is needed in order to do the study. Your permission to use and share your health information has no time limit. You may cancel your decision to share your health information at any time. If you cancel it, you will not be able to stay in this study. Once you have cancelled, no new information or new biological samples (for example, blood) will be collected from you. If you cancel, the study team will still be allowed to use the information that they have already collected from you.

To cancel your permission, you will need to send a letter to Dr. David Diemert stating that you are canceling your permission. This letter must be signed and dated and sent to this address:

Dr. David Diemert
George Washington University
2300 Eye Street NW, Ross Hall Room 524
Washington, DC 20037

A description of this clinical trial will be available on http://www.ClinicalTrials.gov, as required by US law. This website will not include information that can identify you. At most, the website will include a summary of the results. You can search this website at any time. We may also publish the results from this study in journals or present it at scientific meetings. If we do, we will not use your name.

WHAT ARE THE POSSIBLE BENEFITS OF TAKING PART IN THIS STUDY?

There will be no direct benefit to you for taking part in this study. However, your participation may help us to develop a vaccine for hookworm and could help people all over the world in the future.

WHAT OTHER OPTIONS ARE THERE?

An alternative would be to not participate in this study. You do not have to participate in this study, and no one will be upset with you if you decide you do not want to take part.

ARE THERE ANY PAYMENTS TO YOU FOR TAKING PART IN THE STUDY?

You will receive $30 for every study visit to the clinic that you complete. This will cover your time, inconvenience, and travel expenses to and from the study site. In addition, a bonus of $100

will be paid to you at the last visit if you complete all study visits. You will receive a total of $640 if you complete the entire study.

Your name and social security number will be reported to the appropriate George Washington University employees for purposes of making and recording the payment. You are responsible for paying income taxes on any payments provided by the study. If the payments total $600 or more, the University must report the amount you receive to the Internal Revenue Service (IRS) on the form 1099-Misc. This form tells the IRS that payment was made to you, but it does not say that you were paid for taking part in this research study. You should talk to your tax advisor regarding the proper use of this form 1099-Misc.

WHAT ARE THE COSTS?

All research tests and procedures will be paid for by the sponsor. Neither you nor your health insurance company will be charged for the cost of any research tests or procedures that are being done as part of this research study.

WHAT HAPPENS IF YOU GET INJURED OR NEED EMERGENCY CARE?

It is important that you tell someone on the study team if you feel that you have been injured because of taking part in this study. You can tell someone on the study team in person or call Dr. Diemert at 202-270-2393.

If you get hurt or sick from participating in the study*,* you should immediately contact the study team. We will give you any urgent medical treatment needed if the injury is reported in a timely manner. The George Washington Medical Faculty Associates may seek payment from your health insurance company or other third-party payor for any medical care or services you receive. Some health care plans may not cover the costs associated with treating an injury that may result from your participation in this research. If your health insurance plan does not pay the costs associated with treating such an injury, you may be responsible for the payment. However, the sponsor of the research study (the Sabin Vaccine Institute) has an insurance policy that will pay for these costs if they are determined to be related to the research.

The George Washington Medical Faculty Associates, the George Washington University and the George Washington Hospital will not provide you with financial payments for, or related to your injury. No financial compensation will be provided for such things as lost wages, disability or discomfort, losses claimed by spouses or family members, medical expenses due to treatment of any underlying or unrelated condition, or any expense arising from or claimed to be due to any research-related injury. By signing this form you have not given up any of your legal rights.

WILL YOU RECEIVE NEW INFORMATION ABOUT THIS STUDY?

During the study, we may learn new things about the risks or benefits of being in the study. If we do, we will share this information with you. You might change your mind about being in the study based on this information.

WHAT ARE YOUR RIGHTS AS A PARTICIPANT, AND WHAT WILL HAPPEN IF YOU DECIDE NOT TO PARTICIPATE?

Your participation in this study is voluntary. Your decision whether or not to take part will not affect your current or future care at the George Washington Medical Faculty Associates or the George Washington University Hospital. You are not giving up any legal claims or rights. If you decide to take part in this study, you are free to change your mind and stop being in the study at any time. If you are a student or employee at George Washington University, your academic standing/employment status will not be affected in any way should you choose not to take part or to withdraw at any time.

CAN YOU BE REMOVED FROM THE STUDY?

You may be removed from this study without your consent if you do not follow the study team's instructions, at the discretion of the study doctors or the sponsor, or if the sponsor closes the study.

WHOM DO YOU CALL IF YOU HAVE QUESTIONS OR CONCERNS?

You may contact Dr. David Diemert, MD, at 202-994-2909 or 202-270-2393 with any questions, concerns, or complaints about the research or your participation in this study or if you feel you have been hurt by taking part in this study. If you have questions, concerns, or complaints about the research and are unable to contact the study team, contact the George Washington University's Institutional Review Board (IRB) Office at 202-994-2715 between the hours of 9:00 AM and 5:00 PM, Monday to Friday. (Fax: 202-994-0247 or email at ohrirb@gwu.edu).

If you have any questions about your rights as a research participant, or want to talk to someone independent of the research team, you may contact the Institutional Review Board Office at the numbers above.

The IRB is a research review board that is made up of professionals and community members who review and monitor research studies to protect the rights and welfare of research participants.

You will get a copy of this consent form.

CHOICES FOR BLOOD SAMPLES COLLECTED AS PART OF THIS RESEARCH:

Please indicate below by writing your initials your permission of using your blood samples for future research.

My blood may be kept for use in future medical research related to hookworm or immune responses to vaccines.

Yes ________ No ________ (*Participant initials*)

My blood may be shared with other researchers conducting research related to hookworm or immune responses to vaccines.

Yes ________ No ________ (*Participant initials)*

AGREEMENT:

If you want to take part in this study, please sign below.

____

Name of Research Participant	Signature	Date Signed

AFFIDAVIT OF PERSON OBTAINING CONSENT: I certify that I have explained to the above individual(s) the nature and purpose of the study, potential benefits, and possible risks associated with participation in this study. I have answered any questions that have been raised.

____

Name of Person Obtaining Informed Consent	Signature	Date Signed
